# Association of Traditional Chinese Medicine Therapy with Risk of Total Hip Replacement in Patients with Nontraumatic Osteonecrosis of the Femoral Head: A Population-Based Cohort Study

**DOI:** 10.1155/2019/5870179

**Published:** 2019-02-24

**Authors:** Yu-An Yeh, Jen-Huai Chiang, Mei-Yao Wu, Chun-Hao Tsai, Horng-Chaung Hsu, Hsin-Cheng Hsu, Tsung-Li Lin

**Affiliations:** ^1^Chinese Traumatology, Department of Chinese Medicine, China Medical University Hospital, Taichung, Taiwan; ^2^Management Office for Health Data, China Medical University Hospital, Taichung, Taiwan; ^3^College of Medicine, China Medical University, Taichung, Taiwan; ^4^Chinese Internal Medicine, Department of Chinese Medicine, China Medical University Hospital, Taichung, Taiwan; ^5^Department of Orthopedics, China Medical University Hospital, Taichung, Taiwan; ^6^Graduate Institute of Clinical Medicine, China Medical University, Taichung, Taiwan; ^7^School of Post-Baccalaureate Chinese Medicine, College of Chinese Medicine, China Medical University, Taichung, Taiwan; ^8^Department of Sports Medicine, China Medical University, Taichung, Taiwan

## Abstract

**Background:**

Osteonecrosis of the femoral head (ONFH) contributes to 45% of total hip replacements (THRs) annually in Taiwan. Nontraumatic ONFH (NONFH) is multifactorial; no effective Western medicine is available to delay the disease process. This population-based cohort study investigated the association of traditional Chinese medicine (TCM) therapy with risk of THR in patients with NONFH.

**Methods:**

This retrospective study was conducted using claims data from all insured residents covered by the National Health Insurance from 2000 to 2010. We enrolled 1,680 newly diagnosed ONFH patients who had not undergone THR, before or within 6 months after diagnosis of ONFH; these patients did not exhibit hip fracture or dislocation before the endpoint. In total, 595 propensity score-matched pairs were selected from among 1,028 TCM users and 652 non-TCM users. The association between TCM use and risk of THR was analyzed using a Cox proportional hazard model. Kaplan-Meier and log rank tests were performed to plot the cumulative incidence of THR.

**Results:**

The mean follow-up periods were 5.00 years and 3.57 years for TCM and non-TCM cohorts, respectively. Compared to the non-TCM cohort, the TCM cohort had fewer patients undergoing THR surgery (25.4% vs. 18.2%, adjusted hazard ratio: 0.60,* p*<0.0001). The risk of reduction was noted in the group aged 30–59 years (adjusted hazard ratio: 0.56,* p*<0.0001), but there was no association with gender nor socioeconomic status. There was a significantly lower cumulative incidence of THR in TCM users (*p*<0.0001). Shu-Jing-Huo-Xue-Tang and Yan Hu Suo were the most frequently prescribed formula and single herb, respectively.

**Conclusions:**

NONFH patients using TCM had a lower risk of THR; the risk of reduction was noted in the group aged 30–59 years but was not associated with gender nor socioeconomic status. TCM might be useful in conservative treatment for NONFH.

## 1. Introduction

Osteonecrosis of the femoral head (ONFH) is a major worldwide public health concern. Annually, there are 10,000 to 20,000 new cases reported in the United States [1] and 100,000 to 200,000 new cases reported in China [2]. The annual incidence of ONFH as an indication for total hip replacement (THR) has been reported as relatively low in the United States (10%) [3] and the United Kingdom (2%) [4]. Conversely, ONFH has been reported as a major indication for THR in Asian populations: 50% in Korea [5], 49% in India [6], 45% in Taiwan [7], 42% in Singapore [8], and 41.2% in Hong Kong [9].

ONFH is defined as bone cell death due to interruption of the blood supply to the femoral head [10]. Typical ONFH occurs after physical trauma [11], whereas the etiology of nontraumatic ONFH (NONFH) is complex and multifactorial [12]. Steroid usage, alcohol consumption, autoimmune disease (e.g., systemic lupus erythematosus, vasculitis, and rheumatoid arthritis), diabetes, hyperlipidemia, renal failure, dialysis, pancreatitis, and hematologic diseases are all associated with increased risk of NONFH [12–16]. In Taiwan, alcohol consumption is the most prevalent etiology (45.2%), followed by idiopathic causes (33.1%) and steroid usage (21.7%) [17].

Treatment of ONFH is stage-dependent [11]. Notably, many nonsurgical management strategies, such as Western pharmacological management strategies (lipid-lowering agents, anticoagulants, vasoactive substances, and bisphosphonates) and biophysical treatments (extracorporeal shockwave therapy [ESWT], pulse electromagnetic therapy [PET], and hyperbaric oxygen therapy [HBO]) have been found to improve pain and functional outcomes in the early stage [18]. However, most Western conservative therapies to prevent the onset of THR and delay its progression are controversial and lack sufficient evidence for widespread use [11, 18]. Surgical treatment, such as core or multiple drilling decompression in the early stage of NONFH, may be effective in relieving symptoms; however, this approach has no greater value than conservative management in preventing collapse of the femoral head [12, 19, 20].

Traditional Chinese medicine (TCM) has been widely applied in Asia and reportedly provides pain relief and improved quality of life in NONFH patients [21, 22]. However, no evidence has yet indicated that TCM could be useful in the prevention of THR. Therefore, the aim of this nationwide population-based retrospective cohort study was to investigate the association between use of TCM therapy and risk of THR in patients with NONFH.

## 2. Materials and Methods

### 2.1. Data Sources

In Taiwan, TCM has been covered under the government-run National Health Insurance (NHI) program since 1995. This program ensures > 99% of citizens and is accepted by > 93% of healthcare institutes [23]. The NHI research database (NHIRD) provides ambulatory care, inpatient care, and management and medication data and is an ideal platform for use in pharmacoepidemiologic studies. Notably, it comprises a random sample that is representative of the general population; moreover, it includes registration files and original claims data for reimbursements, thereby avoiding selection bias and providing researchers with a comprehensive understanding of healthcare utilization, including Western medicine and TCM treatments [24, 25]. All diseases in the NHIRD are classified in accordance with the International Classification of Diseases, Ninth Revision, and Clinical Modification (ICD-9-CM). For this study, we used the Longitudinal Health Insurance Database 2000 (LHID2000), which comprises all original claims data for 1 million individuals randomly selected from among all beneficiaries of the NHID.

### 2.2. Study Population

**Figure 1:**
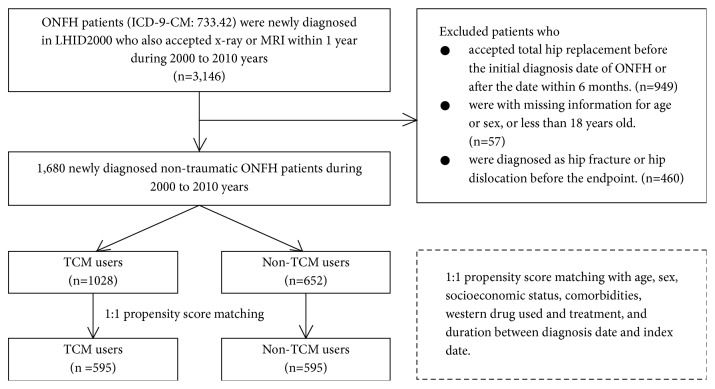
A flowchart about the selection of study subjects.

**Figure 2:**
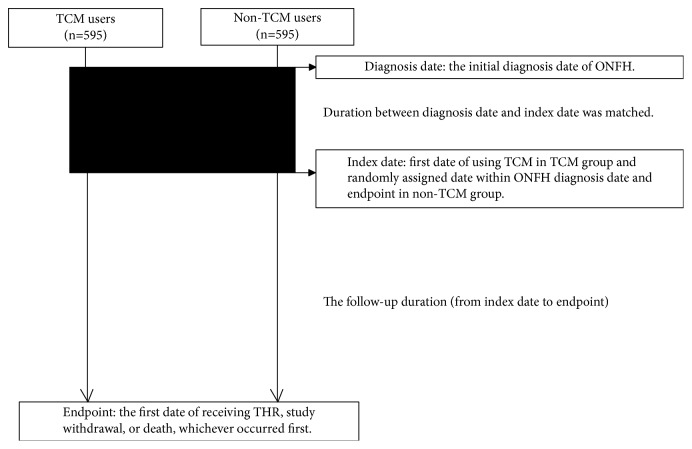
The definitions of diagnosis date, index date, and endpoint.

From January 1, 2000, to December 31, 2010, a total of 3,146 patients were newly diagnosed with ONFH (ICD-9-CM: 733.42) and underwent radiography or MRI within 1 year (Figure 1). We excluded patients who (1) underwent THR before the initial diagnosis of ONFH or within 6 months after the date of initial diagnosis (n=949), (2) had records in which data were missing regarding age or sex, or who were less than 18 years of age (n=57), and/or (3) were diagnosed with hip fracture or hip dislocation (ICD-9-CM: 733.14-15, 820.xx, 821.xx, 905.3, 718.25, 718.28-9, 718.3x, and 754.3x, 835.xx) before the endpoint (n=460). Of the remaining 1,680 patients with NONFH, those with at least one medical record in the TCM outpatient clinic were defined as TCM users (n=1,028) and those with no TCM outpatient records were defined as non-TCM users (n=652). For each TCM user, a control subject who was a non-TCM user was randomly selected by propensity score matching, according to age, sex, socioeconomic status, comorbidities, Western drugs used, surgical treatments, and the duration between the diagnosis date and the index date. Diagnosis date was the initial date of NONFH diagnosis. Index date was the first date of TCM use in the TCM group, whereas it was a randomly assigned date between the diagnosis date and the endpoint (see “Primary Outcome” subsection for details) in the non-TCM group (Figure 2). Finally, 595 propensity-matched pairs of TCM and non-TCM patients were used in this analysis.

### 2.3. Primary Outcome

The primary outcome was THR during the study period (2000 to 2013). The endpoint was the date of THR, study withdrawal, or death, whichever occurred first (Figure 2).

### 2.4. Covariate Assessment

Sociodemographic factors assessed in this study included age, sex, and socioeconomic status. Age was divided into three groups: 18–29 years, 30–59 years, and ≥60 years. Sex was categorized as male or female. Socioeconomic status was divided into two groups according to monthly salary: fixed premium and dependent (<20,000 New Taiwan Dollar, NTD; 1 USD = 30 NTD) and ensured salary grading above fixed premium (≥20,000 NTD). Baseline comorbidities were considered present if the following ICD-9-CM codes appeared in at least three outpatient claims or one inpatient claim before the initial date of ONFH diagnosis: alcohol-related disease (ICD-9-CM: 291, 303, 303.0, 303.00-03, 303.9, 303.90-93, 305.0, 305.01-03, 357.5, 425.5, 535.3, 535.30-31, 571.0-3, 790.3, 977.3, 980.0, and 980.8-9); autoimmune disease: lupus erythematosus (ICD-9-CM: 695.4, 373.34, 710.0), vasculitis (ICD-9-CM: 136.1, 443.1, 446.0-2, 446.4-5, and 446.7), and rheumatoid arthritis (ICD-9-CM: 714.0); diabetes (ICD-9-CM: 250), hyperlipidemia (ICD-9-CM: 272.0-5, 272.7-9); pancreatitis (ICD-9-CM: 577.0-1); hematologic disease (ICD-9-CM: 205, 238.4, 282, 284, and 286); and renal failure or dialysis (ICD-9-CM: 584-586, 403.01, 403.11, 403.91, 404.02, 404.12, and 404.92; treatment code: 58001C, 58027C, 58029C, and 58002C). Western medicines (e.g., bisphosphonate, anticoagulants, iloprost, statins, oral steroids, or intravenous steroids) and surgical treatments (e.g., cord decompression, bone graft, and osteotomy) between initial diagnosis date and endpoint were also considered.

### 2.5. Statistical Analyses

Differences between the two groups were assessed using the chi-squared test or Fisher's exact test for categorical variables and by Student's t-test for continuous variables. The Cox proportional hazard model with hazard ratios (HR) and 95% confidence intervals (CI) was used to estimate the association between TCM use and the risk of THR, as well as the risks between TCM use and each of multiple covariates among NONFH patients. Kaplan-Meier analysis and log rank tests were performed to plot the cumulative incidence of THR. A *p* value <0.05 was considered to be statistically significant. All analyses were performed using SAS statistical software (version 9.4 for Windows; SAS Institute, Inc., Cary, NC, USA).

### 2.6. Ethical Consideration

All patient information that could be used to identify individuals or care providers was deidentified and encrypted before release. The study was approved by the research ethics committee of China Medical University and Hospital (CMUH104-REC2–115(CR-2)).

## 3. Results

There were no statistically significant differences in age, sex, socioeconomic status, most comorbidities (except autoimmune disease), Western drug use, and surgical treatments between the non-TCM and TCM groups (Table 1). Most patients were male in both groups (73.28% vs 73.78%). The 30–59-year-old group comprised the largest proportion in both groups (65.55% vs. 64.87%). Nearly 60% of patients had income <20,000 NTD per month (fixed premium and dependent group). Alcohol-related disease was the most common comorbidity, followed by hyperlipidemia and diabetes. Before the endpoint, 90.42% of non-TCM users and 90.76% of TCM users had used oral or intravenous steroids. The mean (median) follow-up periods (from index date to endpoint) were 5.00 (4.38) years and 3.57 (3.35) years for the TCM and non-TCM cohorts.

In the TCM cohort, fewer patients underwent THR surgery, compared to the non-TCM cohort (18.2% vs 25.4%); moreover, the TCM cohort had a lower adjusted hazard ratio (0.60, 95% CI: 0.46-0.77,* p *< 0.0001), after adjusting for age, sex, socioeconomic status, comorbidities, Western drug use, and surgical treatments (Table 2). Males had a lower risk of THR than females. However, age and socioeconomic status did not affect the risk of THR.

The TCM cohort, compared to the non-TCM cohort, had a significantly lower incidence rate of THR for women and men (Table 3). The TCM cohort also had a significantly lower incidence rate in the 30–59-year-old subgroup. Although there were more THR events in the ≥60-year-old subgroup in the TCM group, the adjusted hazard ratio was equal to 1 (95% CI: 0.58-1.72). However, the TCM cohort had fewer patients undergoing THR surgery regardless of socioeconomic status.

**Figure 3:**
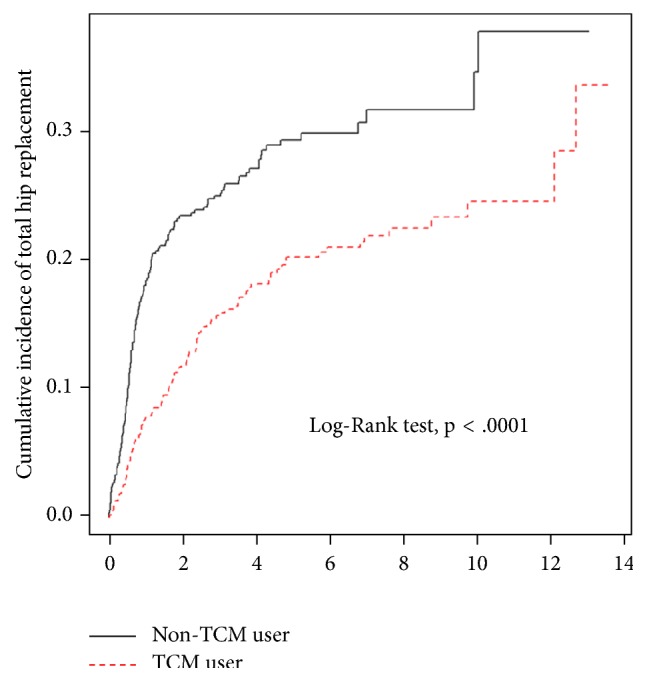
Kaplan-Meier curves of cumulative incidence of THR in patients with NONFH according to TCM use.

Furthermore, Kaplan-Meier analysis with a log rank test showed a significantly lower cumulative incidence of THR in the TCM cohort (*p*<0.0001) than in the non-TCM cohort; the cumulative incidence rate in the TCM cohort remained lower for a follow-up period of up to 12 years (Figure 3).

Table 4 shows the 10 most frequently prescribed formulae and single herbs for treating NONFH in Taiwan. Among multiherb products, Shu-Jing-Huo-Xue-Tang (4,106 person-days) was the most commonly prescribed formula. The herb Yan Hu Suo (4,348 person-days), produced by extraction of a single substance, was the most frequently prescribed single herb.

## 4. Discussion

This study is the first population-based retrospective cohort study regarding the association between TCM use and risk of THR in NONFH patients in Taiwan. Our principal findings were as follows: TCM users had a 0.60-fold lower risk of THR. The risk of reduction was noted in the group aged 30–59 years but was not associated with gender nor socioeconomic status. Furthermore, there was a significantly lower cumulative incidence of THR in TCM users.

TCM has been shown to raise Harris functional scores and improve the quality of life among NONFH patients [21, 22]. However, there has been no large-scale study regarding the potential of TCM to lower the risk of requiring joint replacement surgery. In our study, we enrolled 1,120 NONFH patients, evenly divided into TCM users and nonusers; these patients were matched on the basis of age, sex, socioeconomic status, drugs (including steroids) used, and other treatments that may affect the progression of the disease [12, 17]. We found that male sex and alcohol consumption were prevalent among NONFH patients (Table 1). These results are similar to findings from other studies in Taiwan [17]. The 30–59-year-old age group included 65% of the patients, which was consistent with a previous finding that NONFH occurs most often between the ages of 30 and 50 [26]. We found that nearly 60% of patients with NONFH were fixed premium and dependent group. Because a previous study showed that low-income patients were less likely than their high-income counterparts to undergo total THR [27], we matched the patients on the basis of socioeconomic status (Table 1) and adjusted the analyses based on income (Table 2). These adjusted results show that TCM users had a significantly lower risk of THR, regardless of socioeconomic status (Table 3). Furthermore, TCM users in all age groups had lower incidence rates of THR. However, only in the 30–59-year-old group (65% of patients) had significantly lower risk. The result may be due to the small sample size among patients aged 18–29 and aged ≥60.

NONFH appears to be related to hypercoagulable states, suppression of angiogenesis, hyperadipogenesis, genetic factors, and transition from bone remodeling to bone resorption [16]. Various immune mechanisms and activation of proinflammatory pathways have also been associated with NONFH [12]. The mechanism by which TCM affects NONFH is unclear and may vary on the basis of herbs or formulae used. TCM herb extractions or formulae can reportedly counteract endothelial injury, excessive adipogenesis, blood stasis, and apoptosis; moreover, they can improve osteogenesis, angiogenesis, and blood circulation, both* in vitro* and* in vivo* [28–30]. In the present study, the 10 most commonly prescribed formulae and single herbs were as follows. Shu-Jing-Huo-Xue-Tang (SJHXT) was the most commonly prescribed formulae; this has been shown to relieve muscle pain [31], increase blood circulation [32], and enhance antioxidant enzymatic activity [33]. SJHXT also exhibits anti-inflammatory and analgesic activity [34, 35]. Jia-Wei-Xiao-Yao-San (JWXYS) and Long-Dan-Xie-Gan-Tang (LDXGT) mediate anti-inflammatory activity through inhibition of cytokines and proinflammatory enzymes [36, 37]. However, they have not been shown to improve NONFH. Shao-Yao-Gan-Cao-Tang (SYGCT) has been reported to relieve muscle spasm [38] and spasmodic pain in the musculoskeletal system, including joints, back, and soft tissues [39, 40]. SJHXT and SYGCT can enhance kinase signal pathways, which are important in osteoblast differentiation [41, 42]. Du-Huo-Ji-Sheng-Tang (DHJST) can regulate the expression of vascular endothelial growth factor and hypoxia-inducible factor, inhibit chondrocyte apoptosis [43], promote osteogenic differentiation, and decrease the aging process of human mesenchymal stem cells [44]. These characteristics may be related to the improvement of NONFH. Ge-Gen-Tang (GGT) is named for its major herb composition, Ge-Gen, which was also a commonly prescribed single herb in our cohort study. The major active compound of Ge-Gen, puerarin, reportedly inhibits adipogenic differentiation and prevents alcohol-induced osteonecrosis [29]. Xue-Fu-Zhu-Yu-Tang (XFZYT) is used to enhance blood circulation, counteract blood stasis, and inhibit inflammatory responses and apoptosis [45, 46].

Among single herbs, Yan Hu Suo (YHS) was prescribed most frequently. Tetrahydroprotoberberines, dehydrocorybulbine, and L-tetrahydropalmatine are active components that can be isolated from YHS; these have been demonstrated to exert analgesic and antinociceptive effects on chronic inflammatory and injury-induced neuropathic pain [47–50]. Niu Xi (NX) has been reported to induce angiogenesis and is used as treatment for bone injury [51]; it is a key component in both SJHXT and XFZYT and was commonly used as treatment for NONFH in our study. Moreover, NX is a key component of the Huogu II formula, which showed preventive and therapeutic effects in an experimental model of ONFH [52]. Dan Shen (DS) has lipid-lowering [53] and anti-inflammatory [54] effects; furthermore, it could protect endothelial cells from hydrogen peroxide damage and inhibit apoptosis [55]. Gan Cao (GC) is very popular in TCM and commonly used in herbal formulae to “harmonize” other ingredients; it has anti-inflammatory, antioxidative, and immunomodulatory effects [56, 57].

Each Chinese herb or formula might have different osteogenic, angiogenic, anti-inflammatory, antioxidant, antiadipogenic, and analgesic effects, which contribute to the effect of TCM treatment in reducing the risk of THR among NONFH patients. Overall, TCM treatment might be protective for patients with NONFH.

## 5. Limitations

This study had some limitations. First, we did not evaluate the amount and frequency of steroid or alcohol usage. Second, Western treatments, such as ESWT, PET, HBO, tantalum implants, and biological agents that lack sufficient evidence, were not covered by the NHI program; thus, these were not evaluated. Herbal decoction and folk medicine are also not covered by the NHI program. Therefore, the utilization of TCM may be underestimated. Third, NONFH severity and stage were not assessed. However, we excluded patients who underwent THR before the initial diagnosis date, as well as those who underwent THR within 6 months after the initial diagnosis date. Finally, we did not assess any adverse drug reactions or interactions between Chinese medicine and Western medicine, although these have been previously reported. Despite these limitations, our findings are representative of the general population because we used population-based data from a national database.

## 6. Conclusions

In this retrospective population-based cohort study, TCM users had a lower risk of THR. The risk of reduction was noted in the group aged 30–59 years but was not associated with gender nor socioeconomic status. We also found a significantly lower cumulative incidence of THR among TCM users. However, further prospective studies examining the mechanisms of TCM are needed to confirm our findings.

## Figures and Tables

**Table 1:** Characteristics of NONFH patients according to use of TCM.

Variables	TCM				*p*-value*∗*
	No (n=595)		Yes (n=595)		
	n	%	n	%	
*Sex*					0.8437
Female	159	26.72	156	26.22	
Male	436	73.28	439	73.78	
*Age group*					0.8857
18–29	34	5.71	38	6.39	
30–59	390	65.55	386	64.87	
≥60	171	28.74	171	28.74	
Mean ± SD	51.71 (15.13)		51.61 (14.58)		0.9124 ^a^
*Socioeconomic status*					0.5815
Fixed premium and dependent	359	60.34	348	58.49	
Insured salary grading above fixed premium	236	39.66	247	41.51	
*Comorbidity*					
Alcohol-related disease	387	65.04	384	64.54	0.8555
Autoimmune disease	16	2.69	32	5.38	0.0184
Diabetes	107	17.98	118	19.83	0.4154
Hyperlipidemia	148	24.87	153	25.71	0.7388
Pregnancy	14	2.35	24	4.03	0.0992
Renal failure or dialysis	47	7.9	42	7.06	0.5816
Pancreatitis	19	3.19	13	2.18	0.2823
Hematologic disease	9	1.51	5	0.84	0.2822
*Drug used*					
Bisphosphonate	24	4.03	19	3.19	0.4374
Anticoagulants	103	17.31	112	18.82	0.4977
Iloprost	0	0	0	0	-
Statins	156	26.22	165	27.73	0.5566
Oral Steroid + IV Steroid	538	90.42	540	90.76	0.8426
*Treatment*					
Cord decompression	38	6.39	48	8.07	0.2629
Bone graft (BG)	16	2.69	28	4.71	0.0653
Osteotomy	1	0.17	1	0.17	1.0000^b^
*Duration between diagnosis date and index date (days) (mean, median)*	678 (355)		699 (383)		0.6528 ^a^

*Abbreviations.* NONFH: nontraumatic osteonecrosis of the femoral head; TCM, traditional Chinese medicine.*∗*Chi-squared test; ^a^ t-test; ^b^Fisher's exact test.

The mean (median) of the follow-up period was 5.00 (4.38) years and 3.57 (3.35) years for the TCM user cohort and non-TCM user cohort, respectively.

**Table 2:** Cox model with hazard ratios and 95% confidence intervals for total hip replacement in TCM users and covariates in NONFH patients.

Variable	Total hip replacement	Crude^*∗*^			Adjusted^†^		
	no. (n=259)	HR	(95% CI)	*p*-value	HR	(95% CI)	*p*-value
*TCM use *							
No	151	1.00	reference		1.00	reference	
Yes	108	0.59	(0.46–0.76)	<.0001	0.60	(0.46–0.77)	<.0001
*Sex*							
Female	75	1.00	reference		1.00	reference	
Male	184	0.86	(0.66–1.13)	0.2785	0.56	(0.42–0.75)	<.0001
*Age group*							
18–29	13	1.00	reference		1.00	reference	
30–59	191	1.46	(0.83–2.56)	0.1866	1.31	(0.73–2.35)	0.3739
≥60	55	1.01	(0.55–1.84)	0.9822	1.05	(0.55–2.02)	0.8718
*Socioeconomic status*							
Fixed premium and dependent	144	1.00	reference		1.00	reference	
Insured salary grading above fixed premium	115	1.12	(0.81–1.58)	0.4984	1.13	(0.81–1.59)	0.5226
*Comorbidity*							
Alcohol-related disease	229	4.78	(3.26–6.99)	<.0001	5.13	(3.47–7.58)	<.0001
Autoimmune disease	11	1.00	(0.54–1.82)	0.9873	1.05	(0.57–1.95)	0.876
Diabetes	35	0.7	(0.49–1.01)	0.0533	0.91	(0.61–1.35)	0.6379
Hyperlipidemia	63	0.98	(0.74–1.31)	0.907	1.64	(1.17–2.30)	0.0037
Pregnancy	5	0.57	(0.24–1.39)	0.22	0.53	(0.21–1.30)	0.1631
Renal failure or dialysis	17	1.01	(0.62–1.65)	0.9657	1.08	(0.65–1.81)	0.7603
Pancreatitis	7	1.15	(0.54–2.44)	0.71	0.78	(0.36–1.7)	0.5283
Hematologic disease	4	1.64	(0.61–4.41)	0.3256	1.59	(0.57–4.4)	0.3750
*Drug used*							
Bisphosphonate	6	0.62	(0.28–1.39)	0.2449	0.53	(0.23–1.23)	0.1399
Anticoagulants	30	0.60	(0.41–0.87)	0.0078	0.65	(0.44–0.96)	0.0295
Iloprost	0						
Statins	280	0.46	(0.33–0.64)	<.0001	0.38	(0.25–0.55)	<.0001
Oral Steroid + IV Steroid	224	0.67	(0.47–0.95)	0.0261	0.66	(0.46–0.96)	0.0281
*Treatment*							
Cord decompression	33	2.09	(1.45–3.01)	<.0001	1.74	(1.17–2.60)	0.0065
Bone graft (BG)	10	0.98	(0.52–1.84)	0.9505	0.73	(0.38–1.43)	0.3597
Osteotomy	0	-	-	-	-	-	-

*Abbreviations.* NONFH: nontraumatic osteonecrosis of the femoral head; TCM: traditional Chinese medicine; HR: hazard ratio; CI: confidence interval.

Crude HR^*∗*^ represents relative hazard ratio; adjusted HR^†^ represents adjusted hazard ratio: mutually adjusted for TCM use, sex, age, socioeconomic status, comorbidities, drug used, and treatment in Cox proportional hazard regression.

**Table 3:** Incidence and Cox proportional hazard regression with hazard ratios and 95% confidence intervals for total hip replacement in TCM users and non-TCM users, stratified by sex, age, and socioeconomic status.

Variable	Traditional Chinese Medicine used						Crude HR	Adjusted HR^‡^
	Non-TCM user			TCM User				
	Event	Person years	IR^†^	Event	Person years	IR^†^		
*Total*	151	2098.05	71.97	108	2958.02	36.51	0.59(0.46–0.76)*∗∗∗*	0.60(0.46–0.77)*∗∗∗*
*Sex*								
Female	43	564.26	76.21	32	719.87	44.45	0.63(0.40–1.00)*∗*	0.60(0.37–0.98)*∗*
Male	108	1533.79	70.41	76	2238.15	33.96	0.58(0.43–0.78)*∗∗∗*	0.64(0.47–0.86)*∗∗*
*Age group*								
18–29	10	136.68	73.16	3	205.69	14.59	0.21(0.06–0.77)*∗*	0.35(0.07–1.66)
30–59	115	1420.76	80.94	76	1948.21	39.01	0.57(0.43–0.77)*∗∗∗*	0.56(0.42–0.76)*∗∗∗*
≥60	26	540.61	48.09	29	804.11	36.06	0.86(0.50–1.45)	1.00(0.58–1.72)
*Socioeconomic status*								
Fixed premium and dependent	84	1220.55	68.82	60	1673.11	35.86	0.61(0.44–0.85)*∗∗*	0.58(0.41–0.81)*∗∗*
Insured salary grading above fixed premium	67	973.53	74.55	48	1338.49	39.82	0.63(0.45–0.88)*∗∗*	0.53(0.34–0.83)*∗∗*

*Abbreviations.* TCM: traditional Chinese medicine; † IR: incidence rates, per 1,000 person-years; HR: hazard ratio; CI: confidence interval.

Adjusted HR^ ‡^ represented adjusted hazard ratio: mutually adjusted for TCM use, sex, age, socioeconomic status, comorbidities, drug used, and treatment in Cox proportional hazard regression.

*∗p*<0.01; *∗∗ p*<0.001; *∗∗∗ p*<0.0001.

**Table 4:** Ten most common formulae and single herbs prescribed for patients with NONFH in Taiwan.

Traditional Chinese medicine	Number of person-days	Frequency	Average daily dose (g)	Average duration of prescription (days)
*Formula*				

Shu-Jing-Huo-Xue-Tang	4,106	461	8.6	6.4
Jia-Wei-Xiao-Yao-San	3,926	672	17.9	7.1
Long-Dan-Xie-Gan-Tang	3,687	504	4.1	5.9
Shao-Yao-Gan-Cao-Tang	2,665	397	4.8	6.6
Du-Huo-Ji-Sheng-Tang	2,482	187	3.2	6.6
Ge-Gen-Tang	2,472	340	18.3	8.9
Xue-Fu-Zhu-Yu-Tang	2,364	347	4.3	6.3
Zhi-Gan-Cao-Tang	2,362	260	3.7	9.2
Liu-Wei-Di-Huang-Wan	2,333	210	3.2	7.7
Suan-Zao-Ren-Tang	2,297	268	16.9	8.2

*Single herb*				

Yan Hu Suo (Rhizoma Corydalis)	4,348	643	2.4	6.8
Niu Xi (Radix Achyranthis Bidentatae)	2,670	382	1.6	7
Dan Shen (Radix Salviae Miltiorrhizae)	2,542	521	1.1	4.9
Gan Cao (Radix Glycyrrhizae)	2,525	324	0.8	7.8
Xu Duan (Dipsacus Japonicas)	2,473	324	1.1	7.6
Du Zhong (Eucommia Ulmonoides)	2,371	362	3.1	6.5
Hai Piao Xiao (Os Sepiae Seu Sepiellae)	2,331	301	2.5	7.7
Da Huang (Radix Et Rhizoma Rhei)	2,270	334	0.8	6.8
Ge Gen (Radix Puerariae)	2,184	322	5.1	6.8
Fuzi (Radix Aconiti Lateralis Preparata)	2,167	279	2.7	7.8

*Abbreviations.* NONFH: nontraumatic osteonecrosis of the femoral head.

## Data Availability

The data used to support the findings of this study are included within the article.
